# Relation between intravascular and intraorgan gas volume and quantity via postmortem CT and putrefaction

**DOI:** 10.1007/s12024-025-01029-0

**Published:** 2025-07-09

**Authors:** Maiko Yoshida, Yohsuke Makino, Masatoshi Kojima, Fumiko Chiba, Go Inokuchi, Hirotaro Iwase

**Affiliations:** 1 Department of Legal Medicine, Graduate School of Medicine, Chiba University, 1-8-1 Inohana, Chuo-Ku, Chiba, Chiba Prefecture, Japan; 2 Department of Forensic Medicine, Graduate School of Medicine, The University of Tokyo, 7-3-1, Hongo, Bunkyo-Ku, Tokyo, Japan

**Keywords:** Intravascular gas, Intraorgan gas, Putrefaction, Postmortem CT

## Abstract

This study assessed the relationship between intravascular and intraorgan gas volume and quantity via postmortem computed tomography (PMCT) and putrefaction. Consecutive medico-legal autopsy and PMCT data from January 2018 through December 2020 (*n* = 1,156) at our institution were collected. Cases of injury within deep subcutaneous lesions (*n* = 146), air embolism (*n* = 2), resuscitation (*n* = 369), or severe postmortem changes (*n* = 192) were initially excluded. However, of these 192 cases with severe postmortem changes, fifty-one cases were reincluded for intraorgan gas assessment because their target organs were maintained in the PMCT images for the gas assessment. A total of 498 cases were included for intraorgan gas assessment, whereas 447 cases were included for intravascular gas assessment. The putrefaction level is classified as follows: stage 1 (S1), no visual putrefaction; S2, partial putrefaction; and S3, total putrefaction. Intravascular gas was classified as follows: Grade 0 (G0); no gas, G1; maximum gas diameter within 10 mm, G2; larger than 10 mm; and G3, larger than half of the maximum anteroposterior diameter of the atrium or ventricle. Intraorgan gas was classified as follows: G0, no gas; G1, nodular gas; G2, dendritic gas; and G3, foamy gas. Intravascular and intraorgan gas in each organ increased significantly as putrefaction progressed (p < 0.01). G1 gas in the right heart appeared from S1 in approximately 20% of the cases, and there was no intravascular G2 or G3 gas in S1. Gas in the liver appeared from S1 in approximately 22% of the cases. The amount of G3 gas in each organ increased significantly in S3. PMCT revealed that intravascular and intraorgan gas accumulated as putrefaction progressed. The G2 and G3 gases in S1 were unusual.

## Introduction

Postmortem CT (PMCT) has been used for forensic practice in many facilities worldwide [1–8]. One of the critical roles of PMCT is the noninvasive detection of pathological gas in a dead body, as this gas is related to the cause of death in cases of air embolism and trauma expressing antemortem pathology. Moreover, gas can be detected for other reasons, such as postmortem changes induced by putrefaction and resuscitation [9–14].

Differentiating pathological and nonpathological gases is crucial when detecting and interpreting gas via PMCT. Therefore, understanding the changes in the appearance of intravascular and intraorgan gas in PMCT images on the basis of the putrefaction stage is necessary. However, to our knowledge, no such study has been conducted to date.

This study assessed the relationship between intravascular and intraorgan gas volume and quantity detected via PMCT and putrefaction.

## Materials and methods

### Subjects

**Fig. 1:**
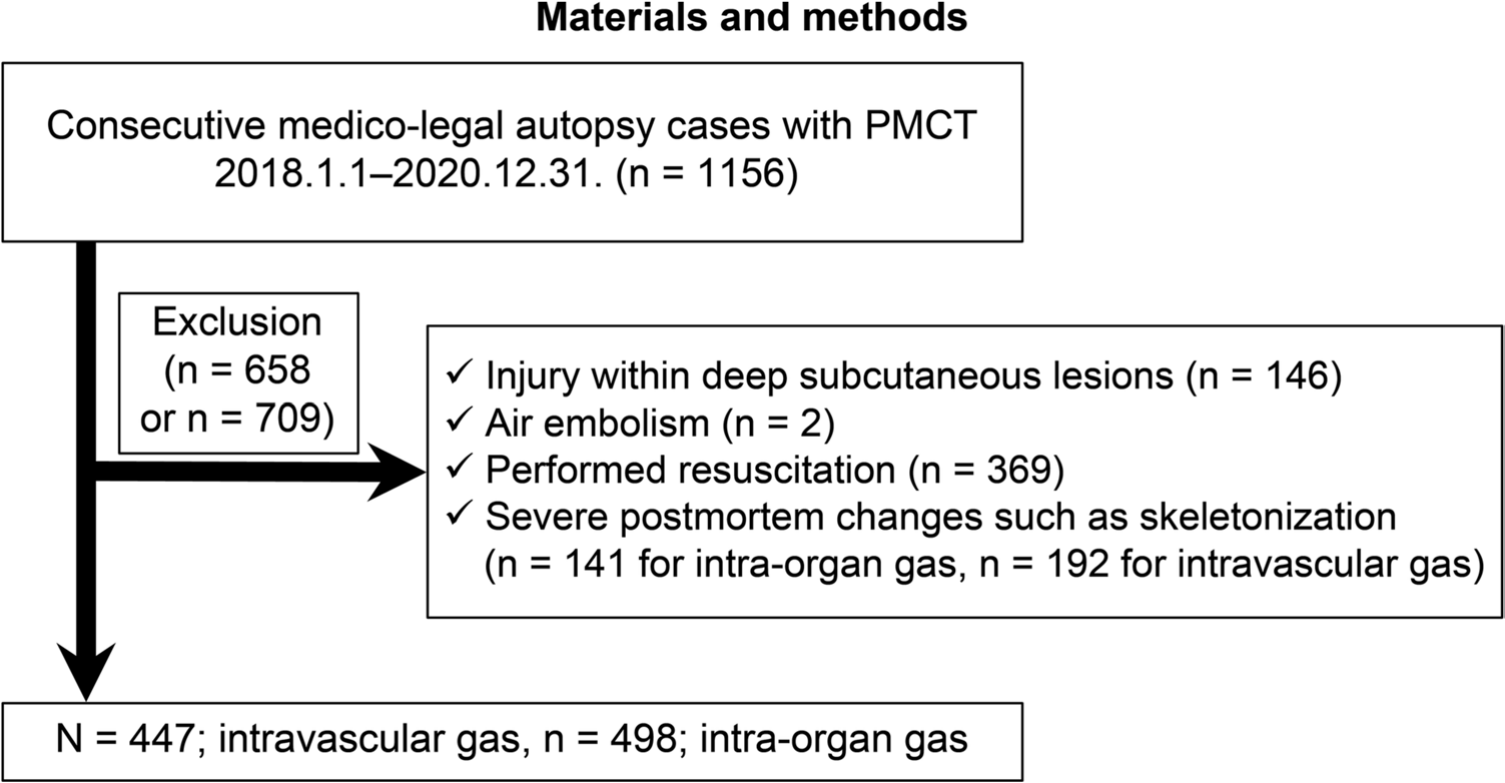
Study groups

**Fig. 2:**
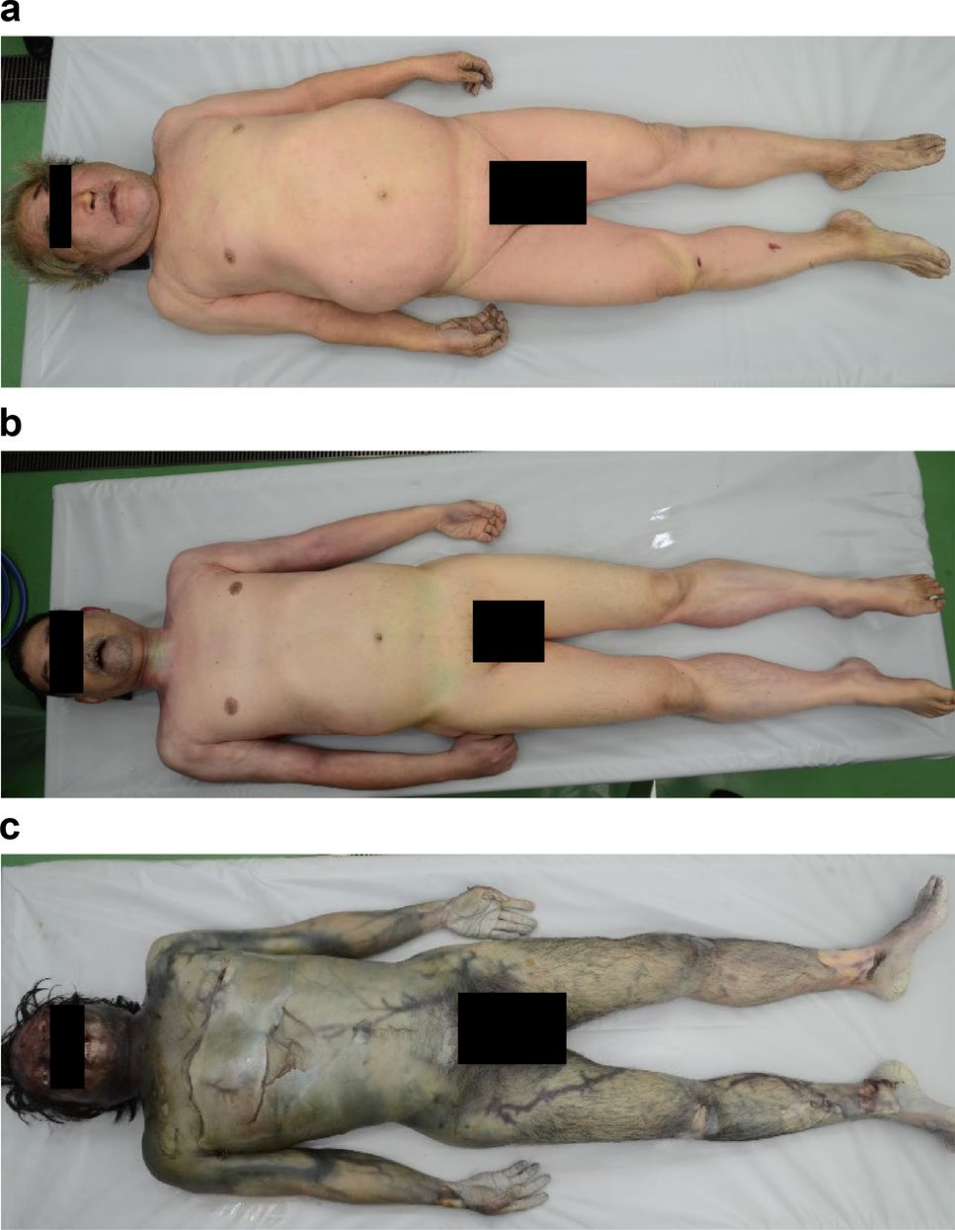
Putrefaction stage grouping; the putrefaction level is classified into three categories: (**a**) stage 1 (S1), no visual putrefaction; (**b**) S2, partial putrefaction; and (**c**) S3, total putrefaction

Consecutive medico-legal autopsy data were collected from January 2018 to December 2020 (*n* = 1,156). Cases with injury within deep subcutaneous lesions (*n* = 146), air embolism (*n* = 2), resuscitation (*n* = 369), or severe postmortem changes (*n* = 192) were initially excluded. However, of these 192 cases with severe postmortem changes, fifty-one cases were reincluded for intraorgan gas assessment because the target organs were maintained in the PMCT images for the gas assessment. A total of 498 cases were included for intraorgan gas assessment, and 447 cases were included for intravascular gas assessment (Fig. 1). Intravascular gas distribution in the right heart, pulmonary artery, and left ventricle and intraorgan gas in the liver, spleen, and kidney were assessed. The putrefaction level was classified as follows: stage 1 (S1), no visual putrefaction; S2, partial putrefaction; and S3, total putrefaction (Fig. 2). A total of 122 cases were classified as S1, 225 cases were classified as S2, and 251 cases were classified as S3.

### Autopsy

The autopsies were performed by nine board-certified forensic pathologists. Dissection, gross examination, microscopic evaluation, and toxicological tests were conducted for each case.

### CT technique

Before the autopsy, all the subjects were imaged with whole-body multidetector CT. We used 64-row multidetector CT (Supria Grande, Fujifilm Co., Tokyo Japan). A whole-body scan was obtained with 0.625 mm collimation. The series was obtained using a tube voltage of 120 kV, a tube current of 250 mA, a pitch of 0.83, and a rotation time of 0.75 s. Images were reconstructed at the CT console to a section thickness of 0.625 mm for the head and neck with a 0.5-mm reconstruction interval and 1.00 mm for the body with a 0.75-mm reconstruction interval via soft-tissue and bone algorithms. After reconstruction, the images were sent to a CT workstation (Vincent, Fujifilm Medical, Tokyo, Japan).

### Image interpretation

The gas density was defined as − 500 HU or less.

**Fig. 3:**
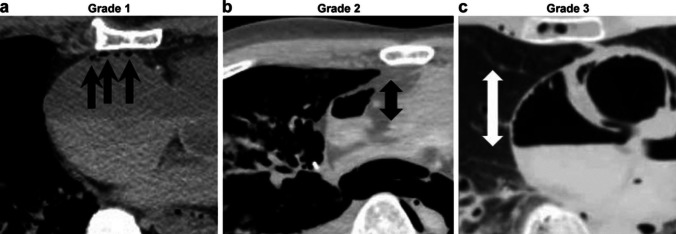
Intravascular gas grouping in the right atrium and both ventricles; four categories were set as follows: grade 0 (G0), no gas; (**a**) G1, maximum gas diameter within 10 mm; (**b**) G2, maximum gas diameter is larger than 10 mm (arrow); and (**c**) G3, maximum gas diameter is larger than half of the maximum anteroposterior diameter of atrium or ventricle (arrow)

**Fig. 4:**
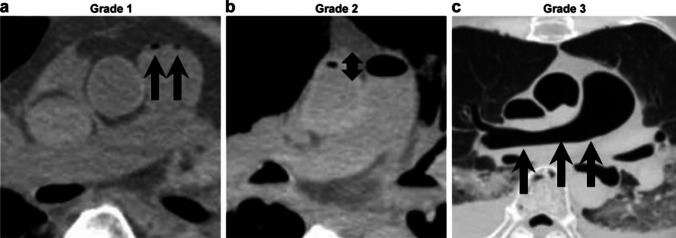
Intravascular gas grouping in the pulmonary artery. Four categories were set as follows: G0, no gas; (**a**) G1, maximum gas diameter within 10 mm; (**b**) G2, maximum gas diameter is larger than 10 mm (arrow); and (**c**) G3, maximum gas diameter crosses the bifurcation of the left and right pulmonary arteries (arrow)

We classified intravascular gas into two systems: the right heart system and the left heart system. *The right heart system* includes the right atrium, right ventricle and pulmonary artery*. The left heart system* consists of the left ventricle. Four Intravascular gas in the right atrium and both ventricles were classified as follows: Grade 0 (G0); no gas, G1; maximum gas diameter within 10 mm, G2; larger than 10 mm; and G3, larger than half of the maximum anteroposterior diameter of the atrium or ventricle (Fig. 3). The gas inside the pulmonary artery was classified as follows: G0, no gas; G1, maximum gas diameter within 10 mm; G2, larger than 10 mm; and G3, maximum gas diameter crossing the bifurcation of the left and right pulmonary arteries (Fig. 4).

**Fig. 5:**
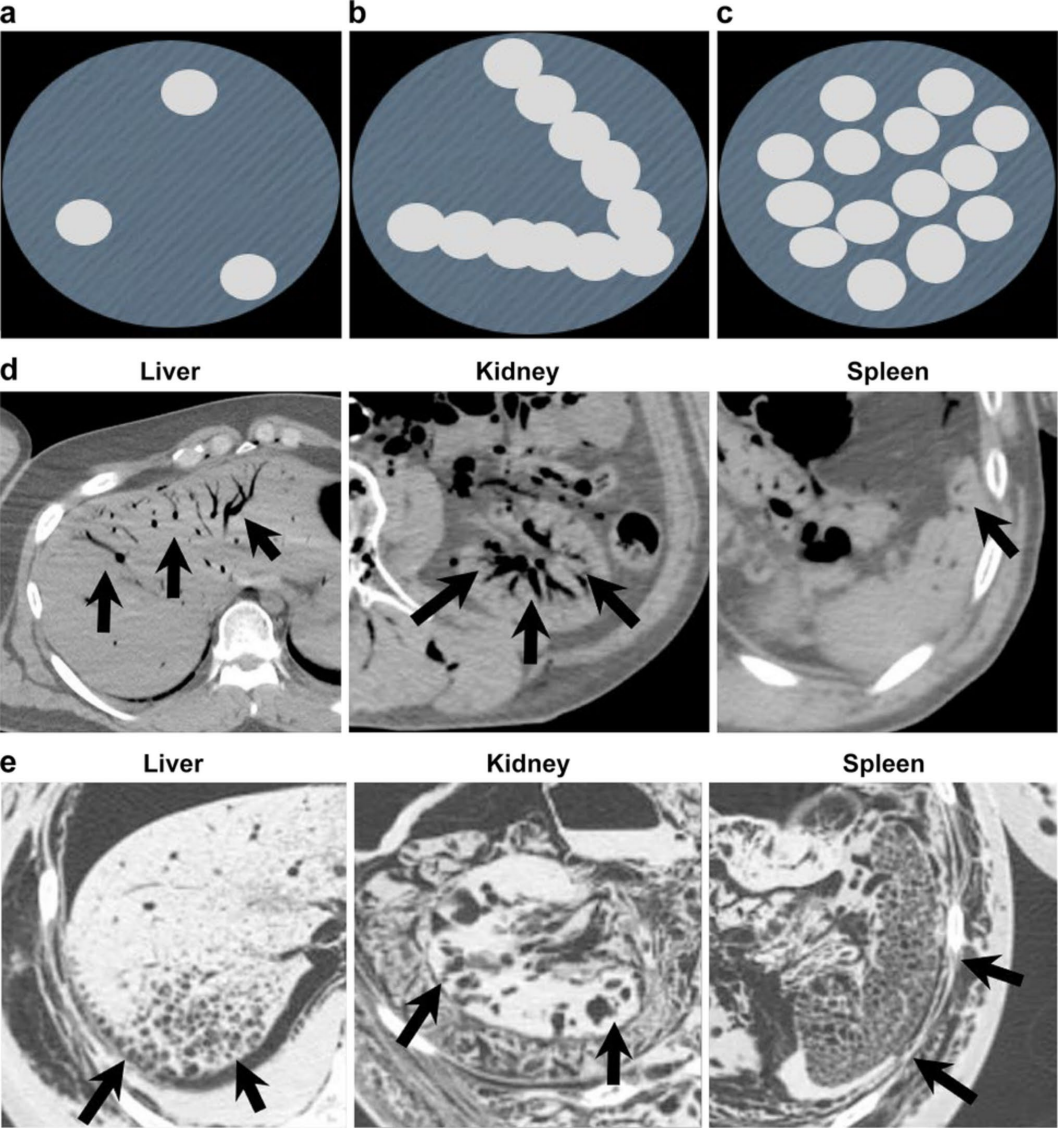
Intraorgan gas grouping. Four categories were set as follows: G0, no gas; (**a**) G1, nodular/scattered gas; (**b**) G2, cord-like/dendritic gas; and (**c**) G3, foamy gas. Actual images of G2 and G3 gas in each organ are shown in (**d**) and (**e**). Detected gas is shown by arrows

For intraorgan gas, the gas density in the liver, kidneys, and spleen was evaluated via PMCT. Intraorgan gas was classified as follows: G0, no gas; G1, nodular/scattered gas; G2, cord-like/dendritic gas; and G3, foamy gas (Fig. 5).

Two board-certified radiologists with 12 years and 6 years of experience in forensic radiology interpreted the PMCT scans. Images were analysed at a workstation with 2D transverse, coronal, sagittal, and oblique datasets and 3D volume-rendered images. The final interpretation was reached by consensus.

### Toxicological analysis

Blood and urine samples were processed for screening by an onsite immunoassay drug screening device combined with instrumental analysis using a 3000 QTRAP LC‒MS‒MS system (SCIEX). Quantitative analysis was performed using the same LC‒MS‒MS/MS system.

### Statistical analysis

Statistical analyses were performed using the chi-square test and Fisher’s test using JMP® 14 (SAS Institute Inc., Cary, NC, USA). A value of *p* < 0.05 was considered statistically significant.

## Results

### Intravascular gas and putrefaction stage

In approximately 20% of cases in the right heart system, G1 gas was detected in S1. Intravascular gas increased as putrefaction progressed in the right and left heart systems. (Table 1).

**Table 1:** Intravascular gas and putrefaction stage: intravascular gas accumulated as putrefaction progressed in the right and left heart systems. In approximately 20% of cases in the right heart system, gas is detected in S1

Putrefaction stage	Right heart system	Left heart system
Stage 1 (*n* = 122)	25/122 (20.4%)	4/122 (3.2%)
Stage 2 (*n* = 225)	72/225 (32.0%)	29/225(12.8%)
Stage 3 (*n* = 151)	100/100 (100%)	86/100(86%)
	*p* < 0.01	*p* < 0.01

A value of *p* < 0.05 was considered statistically significant

#### Gas grade in S1 (*n* = 122)

**Fig. 6:**
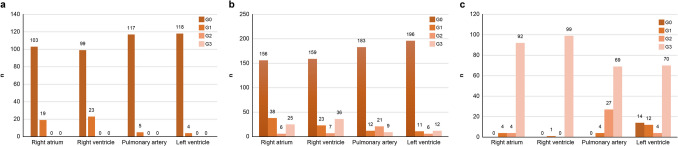
Results of intravascular gas; (**a**) Intravascular gas grade in S1 (*n* = 122), no case showed G2 and G3 gas in the right and left heart systems; (**b**) Intravascular gas grade in S2 (*n* = 225), G2 and G3 gas is detected more frequently than in S1; (**c**) Intravascular gas grade in S3 (*n* = 100), G3 gas increases in S3 both in the right and left heart systems

No case showed G2 and G3 gas in the right or left heart system (Fig. 6 (a)).

#### Gas grade in S2 (*n* = 225)

G2 and G3 gases were detected more frequently than in S1 (Fig. 6 (b)).

#### Gas grade in S3 (*n* = 100)

G3 gas increased significantly in S3 in the right and left heart systems (Fig. 6 (c)).

### Intraorgan gas and putrefaction stage

In the liver, gas was detected from S1 in approximately 22% of the cases. The amount of gas in each organ increased significantly as putrefaction progressed. (Table 2).

**Table 2:** Intraorgan gas and putrefaction stage: In approximately 22% of the cases, gas in the liver appeared from S1. Gas in each organ accumulated as putrefaction proceeds

Putrefaction stage	Liver	Spleen	Kidney
Stage 1 (*n* = 122)	28/122 (22.9%)	2/122 (1.6%)	4/122 (3.2%)
Stage 2 (*n* = 225)	77/225 (34.2%)	23/225 (10.2%)	38/225(16.8%)
Stage 3 (*n* = 151)	151/151(100%)	123/151 (81.4%)	131/151(86.7%)
	*p* < 0.01	*p* < 0.01	*p* < 0.01

A value of *p* < 0.05 was considered statistically significant

#### Intraorgan G2 gas and putrefaction

**Fig. 7:**
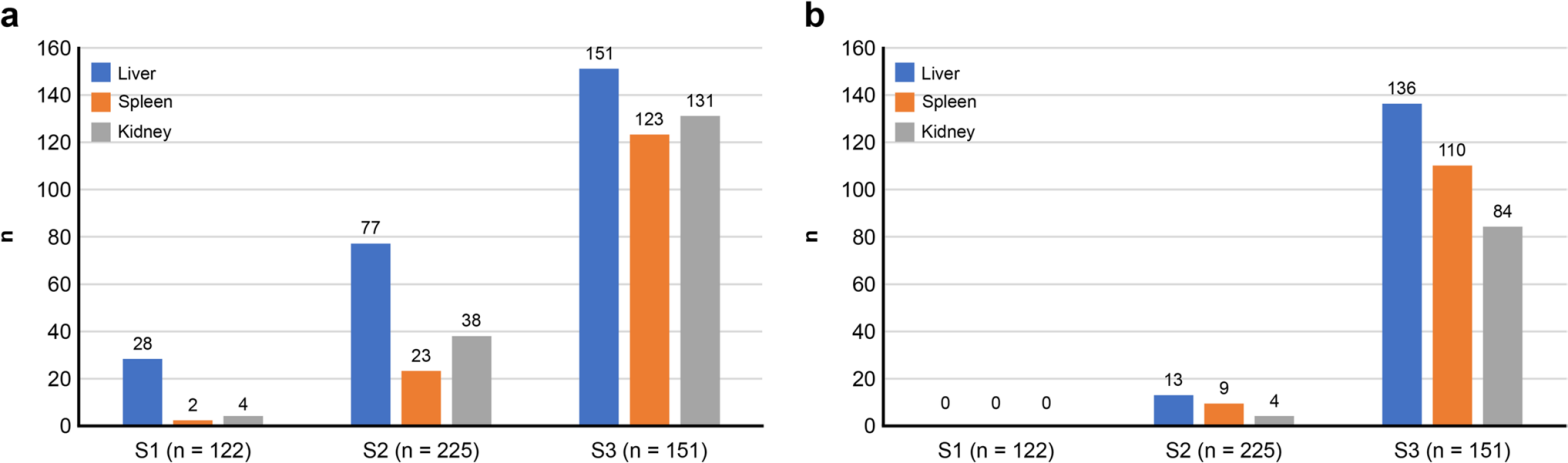
Results of intraorgan gas; (**a**) G2 gas in the liver, spleen and lidney increases as putrefaction proceeds; (**b**) G3 gas in each organ increases significantly in S3

G2 gas in the liver, spleen and kidney increased as putrefaction progressed (Fig. 7 (a)).

#### Intraorgan G3 gas and putrefaction

G3 gas in each organ increased significantly in S3 (Fig. 7(b)).

## Discussion

The current study revealed the relationship between putrefaction and intravascular and intraorgan gas detected via PMCT. The amount of intravascular and intraorgan gas in each organ increased significantly as putrefaction progressed according to PMCT images. G1 gas in the right heart appeared from S1 in approximately 20% of the cases, and there was no intravascular G2 or G3 gas in S1. Gas in the liver appeared from S1 in approximately 22% of the cases, and the amount of G3 gas in each organ increased significantly in S3.

According to our results for intravascular gas, G2 and G3 gas in the right and left heart systems is rare in cases of putrefaction category S1. G2 and G3 gas in the right and left heart systems could indicate a hidden pathological and nonpathological condition, including air/gas embolism. Furthermore, G2 and G3 intravascular gas in stages S2 and S3 alone do not indicate air/gas embolism.

According to the intraorgan gas assessment, the G3 gas detected in S1 or S2 was abnormal and might indicate an antemortem pathological condition such as an infectious disease. G2 gas in the liver is commonly observed in S1, which does not mean that the findings are caused by putrefaction.

In the interpretation of PMCT findings, gas assessment is crucial. Detecting gas during autopsies is usually difficult. Since gas on PMCT appears for various reasons, including antemortem pathological gas (such as trauma and air embolism) and postmortem putrefaction or resuscitation, its differentiation is critical. Therefore, there have been reports describing this issue [7–13]. One of the highlights of these studies is the findings regarding when and where the postmortem changes begin and how they are expressed on PMCT as gas. Several previous studies have reported that gas usually initially appears at the heart (mainly the right heart system) and liver vessels [14–16]. These findings are compatible with the results of our research. However, to the best of our knowledge, basic research on the relationship between gas findings and the putrefaction stage in this field is lacking; thus, the differentiation of pathological and nonpathological, putrefactive gas remains unclear. Therefore, the strength of this research is that the relationship between gas on PMCT and the putrefaction stage (categorized by visual assessment) was assessed for the first time. The results might provide crucial basic data to refer to when pathological cases such as air embolism are assessed.

There are several limitations in this study. First, air embolism cases were excluded from this research. Those cases might show different gas grades than we expected. Second, the number of cases was relatively small. Finally, we only divided the putrefaction stages into three categories. More detailed classification might be desirable, including the use of scales such as the total body score [17–20].

## Conclusion

The amount of intravascular and intraorgan gas detected via PMCT increased as putrefaction progressed. G2 and G3 gases in S1 are unusual.

### Key points

1. PMCT revealed that intravascular and intraorgan gas accumulated as putrefaction progressed.
2. G1 gas in the right heart appeared from S1 in approximately 20% of the cases.
3. There was no intravascular G2or G3 gas in S1.
4. Approximately 22% of the gas in the liver appeared from S1.

## Declarations

This study was conducted in accordance with the Declaration of Helsinki.
